# The Middle Pleistocene (MIS 12) human dental remains from Fontana Ranuccio (Latium) and Visogliano (Friuli-Venezia Giulia), Italy. A comparative high resolution endostructural assessment

**DOI:** 10.1371/journal.pone.0189773

**Published:** 2018-10-03

**Authors:** Clément Zanolli, María Martinón-Torres, Federico Bernardini, Giovanni Boschian, Alfredo Coppa, Diego Dreossi, Lucia Mancini, Marina Martínez de Pinillos, Laura Martín-Francés, José María Bermúdez de Castro, Carlo Tozzi, Claudio Tuniz, Roberto Macchiarelli

**Affiliations:** 1 Laboratoire AMIS, UMR 5288 CNRS, Université Toulouse III Paul Sabatier, Toulouse, France; 2 Centro Nacional de Investigación sobre la Evolución Humana (CENIEH), Burgos, Spain; 3 Department of Anthropology, University College London (UCL), London, United Kingdom; 4 Centro Fermi, Museo Storico della Fisica e Centro di Studi e Ricerche "Enrico Fermi", Rome, Italy; 5 Multidisciplinary Laboratory, The "Abdus Salam" International Centre for Theoretical Physics, Trieste, Italy; 6 Dipartimento di Civiltà e Forme del Sapere, Università di Pisa, Pisa, Italy; 7 Dipartimento di Biologia Ambientale, Università di Roma "La Sapienza", Rome, Italy; 8 SYRMEP Group, Elettra-Sincrotrone Trieste S.C.p.A., Basovizza (Trieste), Italy; 9 Laboratoire PACEA, UMR 5199, Université de Bordeaux, Bordeaux, France; 10 Centre for Archaeological Science, University of Wollongong, Wollongong, Australia; 11 Laboratoire HNHP, UMR 7194 CNRS, Muséum national d'Histoire naturelle (MNHN), Paris, France; 12 Unité de Formation Géosciences, Université de Poitiers, Poitiers, France; University at Buffalo - The State University of New York, UNITED STATES

## Abstract

The penecontemporaneous Middle Pleistocene sites of Fontana Ranuccio (Latium) and Visogliano (Friuli-Venezia Giulia), set c. 450 km apart in central and northeastern Italy, respectively, have yielded some among the oldest human fossil remains testifying to a peopling phase of the Italian Peninsula broadly during the glacial MIS 12, a stage associated with one among the harshest climatic conditions in the Northern hemisphere during the entire Quaternary period. Together with the large samples from Atapuerca Sima de los Huesos, Spain, and Caune de l’Arago at Tautavel, France, the remains from Fontana Ranuccio and Visogliano are among the few mid-Middle Pleistocene dental assemblages from Western Europe available for investigating the presence of an early Neanderthal signature in their inner structure. We applied two- three-dimensional techniques of virtual imaging and geometric morphometrics to the high-resolution X-ray microtomography record of the dental remains from these two Italian sites and compared the results to the evidence from a selected number of Pleistocene and extant human specimens/samples from Europe and North Africa. Depending on their preservation quality and on the degree of occlusal wear, we comparatively assessed: (i) the crown enamel and radicular dentine thickness topographic variation of a uniquely represented lower incisor; (ii) the lateral crown tissue proportions of premolars and molars; (iii) the enamel-dentine junction, and (iv) the pulp cavity morphology of all available specimens. Our analyses reveal in both samples a Neanderthal-like inner structural signal, for some aspects also resembling the condition shown by the contemporary assemblage from Atapuerca SH, and clearly distinct from the recent human figures. This study provides additional evidence indicating that an overall Neanderthal morphological dental template was preconfigured in Western Europe at least 430 to 450 ka ago.

## Introduction

Together with an isolated deciduous incisor from the site of Isernia La Pineta (Molise), recently dated to 583–561 thousand years (ka), i.e., to the end of Marine Isotope Stage (MIS) 15 [1], and the Ceprano calvarium (Latium), which has been dated between 430 and 385 ka and ascribed to a warm phase of MIS 11 [2–3], the Middle Pleistocene dental remains from Fontana Ranuccio, Latium [4], and Visogliano, Friuli-Venezia Giulia [5], set 450 km apart in central and north-eastern Italy, respectively, represent the oldest human fossil remains of the Italian Peninsula discovered so far [6].

Fontana Ranuccio (FR), discovered in 1972 by A.G. Segre, A. Ascenzi and I. Biddittu [4], is located in the extensional tectonic Anagni intra-Apennine basin of the Latina Valley, 50 km southeast of Rome. The basin had been filled by lacustrine-alluvial sediments which, during the Middle Pleistocene, were covered by pyroclasts from the Alban Hills magmatic province [7]. The site, dated by K-Ar to c. 450 ka [2, 8, 9], has yielded a faunal assemblage that points to a varied environment of forests, clearings, and extensive areas covered by lakes and swamps [8]. Rare handaxes and small flake tools were found in association with four isolated human permanent teeth. This fossil assemblage includes: an upper left canine root (FR3); a lower left lateral incisor (FR2); and two, left and right, mandibular first molars (FR1L and FR1R) [8–11].

Visogliano (Vis.), 18 km northwest of Trieste, first investigated by G. Bartolomei in 1970 [5], consists of two distinct loci, A and B, of a karstic doline resulting from the collapse of an ancient cave. According to the biostratigraphic and archaeological records, the loci are penecontemporaneous. The lower stratigraphic levels, which yielded the human remains, have been dated by electron spin resonance (ESR) and U-series to the time interval between 350 and 500 ka, the level 44 where two tooth specimens are from (Vis. 4 and Vis. 5) having been constrained within the interval 480–440 ka [12, 13]. Sediment depositional processes, faunal spectrum and pollen data suggest deposition during a mild climate phase under humid conditions [14–16]. The lithic assemblage includes discoidal cores, choppers, flakes with a few retouched elements and some protobifaces, mainly flaked on local limestone [12, 15]. The human dentognathic assemblage from Visogliano consists of: a right mandibular fragment (V2) preserving in situ the roots of the P4 and M1, but not their crowns; five isolated maxillary teeth (Vis. 1, Vis. 3, Vis. 4, Vis. 5, Vis. 6); and three likely human tooth fragments (Vis. 7, Vis. 8, Vis. 9) [11, 16–18].

The remains from Fontana Ranuccio and Visogliano sample two human groups that lived in southwestern Europe during MIS 12, at a time approximately corresponding to the early appearance of the Neanderthal clade [19–22]. However, because of the degree of morphological diversity at the macro-regional scale displayed by the European Middle Pleistocene cranial and dental remains, discussions exist about the more likely anagenetic (linear) vs cladogenetic (splitting) evolutionary origin of this clade [19, 22–26].

During the last decade or so, the application of new investigative tools and advanced analytical techniques allowed the noninvasive exploration and high-resolution rendering of subtle aspects of the inner morphology of fossil dental remains [27–31]. With special reference to European Neanderthals, research has revealed a time-related pattern towards the establishment of increasingly derived and unique endostructural tooth features [32–42]. However, in spite of their potential informative value in evolutionary studies, especially in a chrono-geographic context characterized by relatively scarce and scattered remains [22, 43–47], quantitative data on the internal structure and tissue proportions of human dental remains from the European Middle Pleistocene, notably from the MIS 15–10 interval, are still limited [41, 47–50].

With respect to the first record provided by A.G. Segre and A. Ascenzi [8, 9], a later revision of the specimens from Fontana Ranuccio has integrated original aspects of their outer size and morphology considered in a wider comparative context, and has also provided preliminary information on their inner structure (imaged at 330 μm isotropic voxel size by a medical scan system) [10]. Conversely, the structural morphology of the assemblage from Visogliano remains to be detailed [11, 51].

With the aim of identifying the presence, if any, of a Neanderthal-like signature in their inner structure, we applied two- three-dimensional (2-3D) techniques of virtual imaging and geometric morphometrics to the high resolution X-ray microtomography (μCT) record of the tooth remains from Fontana Ranuccio and Visogliano and compared the new body of data to several Middle to Late Pleistocene and extant human specimens/samples from Europe and North Africa. Specifically, depending on the preservation quality of the remains and on the degree of occlusal wear, we comparatively assessed: (i) the crown enamel and radicular dentine thickness topographic variation of the uniquely represented lower incisor (from FR); (ii) the lateral crown tissue proportions of premolars and molars; (iii) the enamel-dentine junction and (iv) the pulp cavity morphology of all available specimens.

## Materials and methods

**Fig 1:**
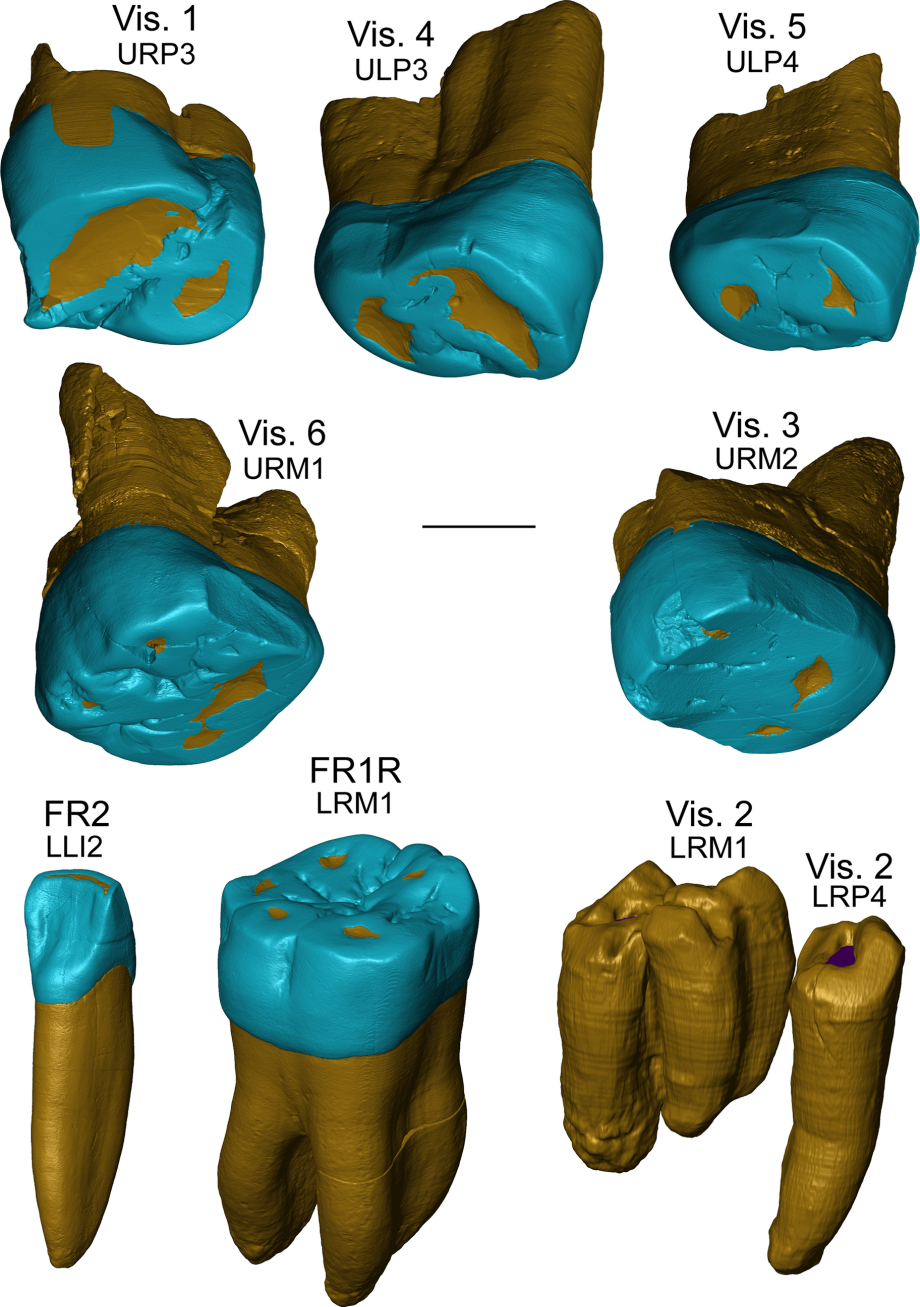
Microtomographic-based rendering of the Fontana Ranuccio (FR1R and FR2) and Visogliano (Vis. 1-Vis. 6) tooth specimens. The enamel is in blue, the dentine in yellow.

We analysed the lower left lateral incisor (LLI2) FR2 and the lower right first molar (LRM1) FR1R from Fontana Ranuccio, and the following five isolated maxillary teeth from Visogliano: the upper right third premolar (URP3) Vis. 1, previously reported as an upper right third molar [11, 17, 18]; the upper left third premolar (ULP3) Vis. 4; the upper left fourth premolar (ULP4) Vis. 5; the upper right first molar (URM1) Vis. 6; and the upper right second molar (URM2) Vis. 3 (Fig 1). We also detailed the Vis. 2 mandibular fragment from Visogliano and, despite its lack of tooth crowns, we virtually extracted the still in situ roots of the right fourth premolar (LRP4) and of the adjacent first molar (LRM1) (Fig 1). Conversely, we did not investigate the isolated FR3 canine root and the LLM1 FR1L specimen from Fontana Ranuccio, nor the isolated tooth fragments Vis. 7, Vis. 8 and Vis. 9 from Visogliano. The moderate occlusal wear and the completely formed roots of FR2 and FR1R are compatible with an attribution to a single adolescent to young adult individual [10]. For Visogliano, the interproximal wear facets of the ULP3 Vis. 4 and the ULP4 Vis. 5, on the one hand, and those of the upper molars Vis. 3 (URM2) and Vis. 6 (URM1), on the other hand, match together. Given the lack in this assemblage of repeated metameric and lateral tooth positions and because of their relative dimensions, morphological features and generalized advanced degree of occlusal wear, the possibility that all teeth are from a single adult individual cannot be rejected in the absence of biogeochemical data [18]. Conversely, at present nothing can be stated about the link between the maxillary elements and the mandibular fragment.

The human fossil dental remains from Fontana Ranuccio (specimens FR2 and FR1R), under the administrative responsibility of the Soprintendeza per i Beni Archeologici del Lazio, Rome (Italy), are permanently stored at the Istituto Italiano di Paleontologia Umana, at Anagni (Frosinone, Italy), while the human fossil assemblage from Visogliano (including the specimens Vis. 1, Vis. 2, Vis. 3, Vis. 4, Vis. 5 and Vis. 6 analyzed here) is permanently curated by the Soprintendenza per i Beni Archeologici del Friuli Venezia Giulia, at Trieste (Italy). All necessary permits were obtained for the described study, which complied with all relevant regulations.

### Microtomographic measurements and image processing and analysis

All specimens were imaged by X-ray microtomography (μCT) at the Multidisciplinary Laboratory of the ICTP [52] and at the Tomolab station of the Elettra Synchrotron light source (http://www.elettra.eu/lightsources/labs-and-services/tomolab/tomolab.html) [53], Trieste, according to the following parameters: 100 to 130 kV voltage, 61 to 90 μA current, and a projection each 0.15° to 0.20°, 2 to 3 mm Al filter. The final volumes were reconstructed using the Shepplogan filter with an isotropic voxel size ranging from 7.8 to 13.6 μm for the isolated teeth, and of 34.1 μm for the mandibular fragment Vis. 2.

Using the commercial software Avizo v.8.0 (Visualization Sciences Group Inc.) and the freeware ImageJ v. 1.51p [54], a semi-automatic, threshold-based segmentation was carried out following the half-maximum height (HMH) method [55] and the region of interest thresholding protocol (ROI-Tb) [56] by taking repeated measurements on different slices of the virtual stack [57], as commonly done on dental tissues [32–42, 47–50]. The 3D surfaces were reconstructed with a constrained smoothing.

Intra- and inter- tests for accuracy of the estimates were run by two observers. Linear, surface, and volumetric measurements provided differences inferior to 4% for both tests.

### Incisor (FR2 [LI2)]) enamel thickness topography and root dentine thickness

The enamel thickness topographic distribution of the LLI2 FR2 specimen from Fontana Ranuccio was rendered through a 3D map generated using a chromatic scale where thickness increases from dark blue (thinner) to red (thicker) [38, 49, 58]. We thus compared the FR2's map to those obtained for the human LRI2 from the late Early-early Middle Pleistocene North African site of Tighenif, Algeria (NAH) [59], a Neanderthal (NEA) specimen from Krapina (KRD), Croatia (MIS 6-5e) [60] and a lateral lower incisor illustrating the most common condition represented in our comparative extant human (EH) European sample. Because of crown size differences among the four lower I2s, we scaled the investigated specimens using the cervical outline as reference. A total of 30 landmarks were placed around their cervix and the three comparative specimens were then warped onto the FR2 surface.

To assess the pattern of root dentine thickness repartition in FR2, we virtually unzipped the 15–85% portion of its total root length along a predefined vertical line on the labial aspect, and then unrolled it and projected its local properties into a morphometric map [38, 49, 61, 62] generated by a custom routine developed in R v.3.4.1 [63] with the packages Momocs [64], spatstat [65] and gstat [66]. For comparative purposes, we applied the same "virtual unrolling" protocol [61] to: the LRI2 from Tighenif [59]; a sample of four Neanderthal lower lateral incisors including the specimens KRD69 and KRD71 from Krapina and both incisors from the partial skeleton Regourdou 1, from the homologous site in Dordogne (France, MIS 4) [49, 58, 60]; and to four extant human LI2s. In the analysis, dentine thickness values have been standardized between 0 and 1 and each morphometric map has been set within a grid of 90 columns and 100 rows. To obtain a consensus map summarizing the signals from the Neanderthal and the extant human representatives, we performed generalized additive models (GAM) of interpolation by merging the individual morphometric maps into single datasets [67–69]. Finally, we used bgPCA for quantitatively assessing the root dentine morphometric properties of the specimen from Fontana Ranuccio with respect to those selected for comparison.

### Premolar (Vis. 1, Vis. 4 [UP3s], Vis. 5 [UP4]) and molar (Vis. 6 [UM1], Vis. 3 [UM2], FR1R [LM1]) lateral crown tissue proportions

Due to their variably worn occlusal surfaces (Fig 1), the 3D assessment of tissue proportions in all upper premolars (Vis. 1, Vis. 4, and Vis. 5) and molars (Vis. 6 and Vis. 3) from Visogliano and in the lower M1 from Fontana Ranuccio (FR1R) was limited to their lateral (non-occlusal) crown portion [70]. Accordingly, a plane parallel to the cervical one and tangent to the lowest enamel point of the occlusal basin was established, all material above it was removed, and only the enamel and dentine portions between these two planes was preserved to estimate tissue proportions [37, 39, 49, 69]. The following parameters were thus calculated on the new set of virtually reduced and simplified crowns: the percent of lateral coronal volume that is dentine and pulp (Vlcdp/Vlc, in %) and the scale-free 3D lateral relative enamel thickness (3D LRET) [37, 39, 49, 69]. The fossil and extant comparative materials used in this analysis include: the North African late Early-early Middle Pleistocene *Homo* from Tighenif [59]; an assemblage of 14 premolars and 22 molars from the Neanderthal sites of La Chaise-de-Vouthon Abri Suard (France, MIS 6), Krapina, and Regourdou [32, 49, 58, 60]; and an extant human European sample of 17 premolar and 19 molar crowns (Tables 1 and 2).

**Table 1:** Fossil and extant human comparative specimens/samples used for assessing premolar and molar 3D lateral crown tissue proportions.

Specimens/Samples	Site/Origin	UP3	UP4	UM1	UM2	LM1	References
North African late Early-early Middle Pleistocene *Homo* (NAH)	Tighenif			Tighenif URM1/2		Tighenif 2	[59]
Neanderthals (NEA)	La Chaise-de-Vouthon Krapina Regourdou	KRD38, KRD39, KRD43, KRD45, KRD48, KRD53, KRD54, KRD55	KRD41, KRD42, KRD44, KRD46, KRD47, KRD49	KRD101, KRD134, KRD136, KRD164, KRD171, KRD174	KRD96, KRD98, KRD135, KRD165, KRD166, KRD169	S14-7, S5, S49, BDJ4C9, KRD77, KRD79, KRD80, KRD81, KRD105, Regourdou 1	[32], [33], [49], [58], [60]
recent humans (RH)		7	7	6	5	8	present study

**Table 2:** 3D lateral crown tissue proportions assessed in the Fontana Ranuccio (FR) and Visogliano (Vis.) premolars and molars and compared with some fossil and extant human specimens/samples representing: North African late Early-early Middle Pleistocene *Homo* (NAH), Neanderthals (NEA) and extant humans (EH). See Table 1 for details on the composition of the comparative samples.

		UP3				UP4				UM1				UM2				LM1	
	N	Vlcdp/Vlc (%)	3D LRET		N	Vlcdp/Vlc (%)	3D LRET		N	Vlcdp/Vlc (%)	3D LRET		N	Vlcdp/Vlc (%)	3D LRET		N	Vlcdp/Vlc (%)	3D LRET
**Vis. 1**	1	74.85	10.42	**Vis. 5**	1	75.67	11.01	**Vis. 6**	1	75.42	11.06	**Vis. 3**	1	76.03	9.13	**FR1R**	1	79.77	8.89
**Vis. 4**	1	77.08	9.99					NAH	1	77.32	9.23	NAH	1	77.32	9.23	NAH	1	79.1	9.32
NEA av.	8	76.13	9.97	NEA av.	6	75.76	10.25	NEA av.	6	77.49	9.86	NEA av.	6	79.94	9.4	NEA av.	10	80.15	8.67
s.d.		3.79	1.83	s.d.		2.35	1.24	s.d.		2.21	0.85	s.d.		5.89	1.49	s.d.		2.78	1.3
min.		70.7	7.99	min.		72.71	8.51	min.		74.82	8.99	min.		74.6	6.63	min.		73.84	6.59
max.		80.33	12.59	max.		79.42	12.16	max.		80.39	10.93	max.		91.43	11.03	max.		85.06	11.65
EH av.	8	70.8	11.66	EH av.	9	70.5	12.43	EH av.	6	75.95	9.79	EH av.	5	79.17	9.45	EH av.	8	78.99	9.31
s.d.		2.5	1.48	s.d.		2.67	1.8	s.d.		1.36	0.56	s.d.		1.83	0.61	s.d.		2.47	1.05
min.		68.27	9.55	min.		67.19	9.61	min.		74.77	8.91	min.		76.24	8.86	min.		76.11	7.54
max.		74.73	13.54	max.		74.11	14.84	max.		78.06	10.28	max.		81	10.39	max.		82.66	10.32

### Enamel-Dentine Junction (EDJ) Morphology (FR2 [LI2], Vis. 1, Vis. 4 [UP3s], Vis. 5 [UP4], Vis. 6 [UM1], Vis. 3 [UM2], FR1R [LM1])

For qualitative observations, we imaged the enamel-dentine junction (EDJ) of all available crowns from Fontana Ranuccio (FR2 and FR1R) and Visogliano (Vis. 1, Vis. 4, Vis. 5, Vis. 6, Vis. 3), independently from their degree of preservation. A comparative GM analysis was uniquely performed on the unsmoothed reconstructed virtual surface of the FR1R lower M1 from Fontana Ranuccio, the specimen preserving the most complete morphology of the occlusal dentine, except for the horn tips. For this analysis, its apices were reconstructed following two independent methods. Firstly, integration was achieved on geometric basis (FR1R geom-rec). To do so, the FR1R virtual slices were resampled to be parallel to the cervical plan. A parallel plane was then shifted towards each dentine horn extremity and two sections perpendicular to the cervical plane, corresponding respectively to the widest mesiodistal and buccolingual diameters of the last section of the dentine horn and intersecting its centre, were used to reconstruct the height and orientation of each apex. Interpolations were then performed for rendering the 3D shape of the tips (S1 Fig). We also reconstructed the dentine horn extremities by using a Neanderthal-like (FR1R NEA-rec) and an extant human-like (FR1R EH-rec) template, respectively. As Neanderthal model we used the LM1 specimen from Fossellone 3, Italy [71, 72]. The EDJs of both templates were superimposed to the original FR1R's shape and the height and orientation of their dentine horn apices were used to create the FR1R NEA-rec and the FR1R EH-rec chimeric models, respectively (S1 Fig). On each of the three reconstructed EDJ outlines (FR1R geom-rec, FR1R NEA-rec and FR1R EH-rec), seven landmarks were placed on the apex of the protoconid, metaconid, entoconid and hypoconid horns, and at each intermediate lowest point between two dentine horns along the dentine marginal ridge, except between the two distal horns and without considering the hypoconulid and the distal marginal ridge (S2 Fig) [59, 62, 73]. Finally, the three versions of the FR1R EDJ were compared by a GPA and a bgPCA analysis to: the LM1 of Tighenif 2 [59], five LM1s from the Middle Pleistocene site of Sima de los Huesos (Spain, late MIS 12 or early MIS 11) [45], a sample of 10 Neanderthal LM1s from the European sites of Ehringsdorf (Germany, MIS 7), La Chaise-de-Vouthon Abri Suard, La Chaise-de-Vouthon Abri Bourgeois-Delaunay (MIS 5e), and Krapina [32, 33, 49, 58, 60], and to 14 extant European human specimens (Table 3).

**Table 3:** Fossil and extant human comparative samples used in the GM analyses of the LM1 EDJ.

Groups	Site/Origin	LM1	References
North African late Early-early Middle Pleistocene *Homo* (NAH)	Tighenif	Tighenif 2*	[59]
Sima de los Huesos (SH)	Sima de los Huesos	AT-829, AT-943, AT-1458, AT-1459, AT-3175	[41], [50]
Neanderthals (NEA)	Ehringsdorf	Ehringsdorf I	[32], [33], [49], [58], [60]
	La Chaise-de-Vouthon	S5, S14-7, S49, BDJ4C9	
	Krapina	KRD77, KRD79, KRD80, KRD81, KRD105	
extant humans (EH)	Europe	14	present study

* Added a posteriori in the bgPCA analysis.

### Pulp cavity morphology (FR2 [LI2], Vis. 1, Vis. 4 [UP3s], Vis. 5 [UP4], Vis. 6 [UM1], Vis. 3 [UM2], Vis. 2 [LP4, LM1], FR1R [LM1])

We virtually extracted and imaged all pulp cavities preserved in the specimens from Fontana Ranuccio (FR2 and FR1R) and Visogliano (Vis. 1, Vis. 4, Vis. 5, Vis. 6, Vis. 3, Vis. 2) investigated in this study, including those that are incomplete. For qualitative comparative evaluation, we considered the available fossil specimens from Tighenif [59] and from Krapina (spec. KRD30, KRD41, KRD45, KRD71, KRD 79, KRD136, KRD165) [60], and a whole of seven extant European teeth.

## Results

### Incisor enamel thickness topography and root dentine thickness

**Fig 2:**
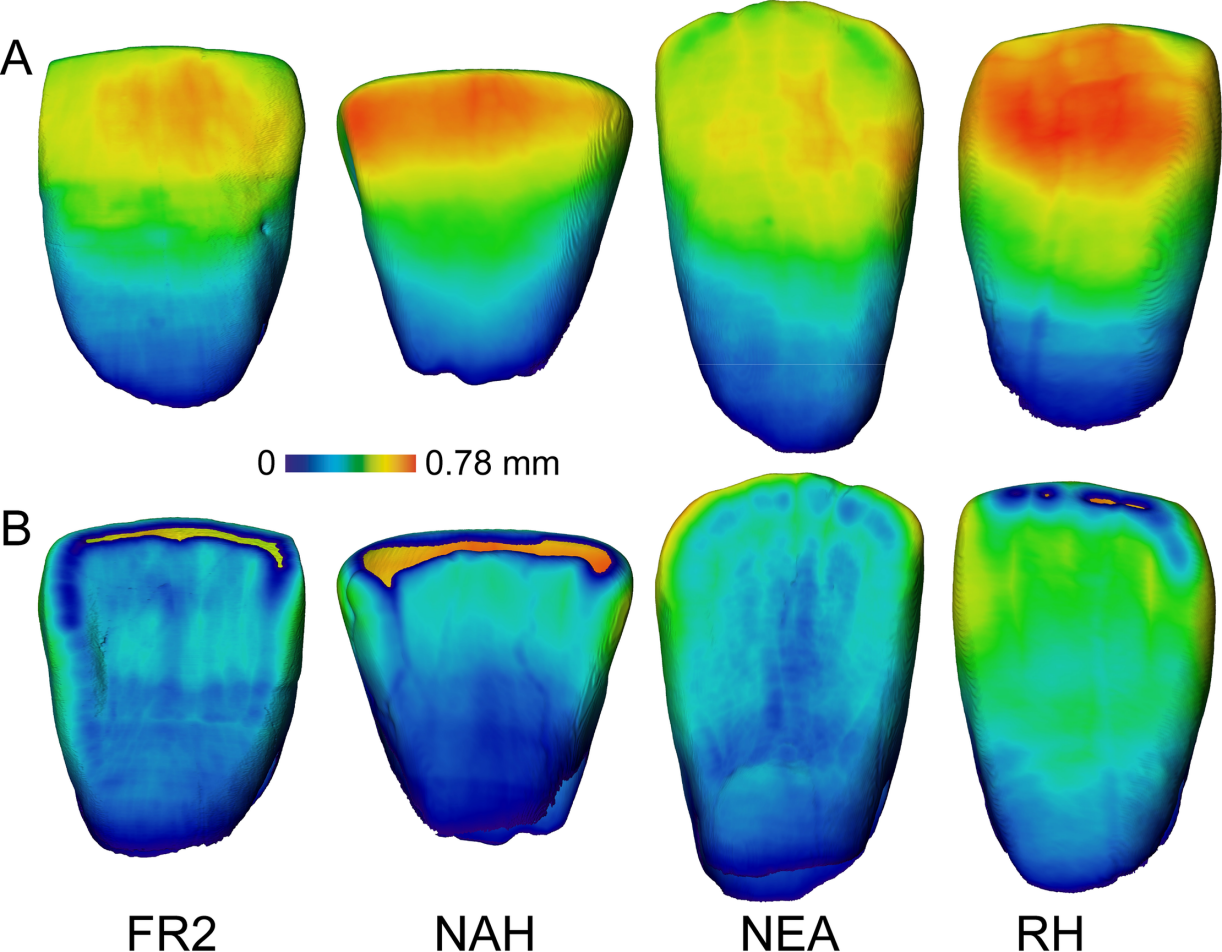
Enamel thickness cartographies of the LLI2 FR2 from Fontana Ranuccio in labial (A) and lingual (B) views compared with similar evidence from the North African late Early-early Middle Pleistocene *Homo* (NAH) from Tighenif [59], the Neanderthal KRD 90 (NEA) [60] and an extant human incisor (original data). For each specimen, topographic variation is rendered by a tooth-specific thickness-related pseudo-colour scale ranging from thinner dark-blue to thicker red.

As seen in labial view, the enamel thickness distribution pattern revealed by the slightly occlusally worn LLI2 FR2 specimen from Fontana Ranuccio is somehow intermediate between that of the late Early-early Middle Pleistocene incisor from Tighenif, whose occlusal crown third is thicker [59], and the typically thinner Neanderthal condition (as here exemplified by the specimen KRD90 from Krapina), the extant human incisor crowns being commonly characterized by absolutely and relatively thick labial enamel (Fig 2A). In all investigated specimens, enamel thickness decreases cervically. However, the cartographies imaging the lingual aspect of the same specimens are less contrasted, but in the extant human representative, which again displays the thickest values (Fig 2B).

**Fig 3:**
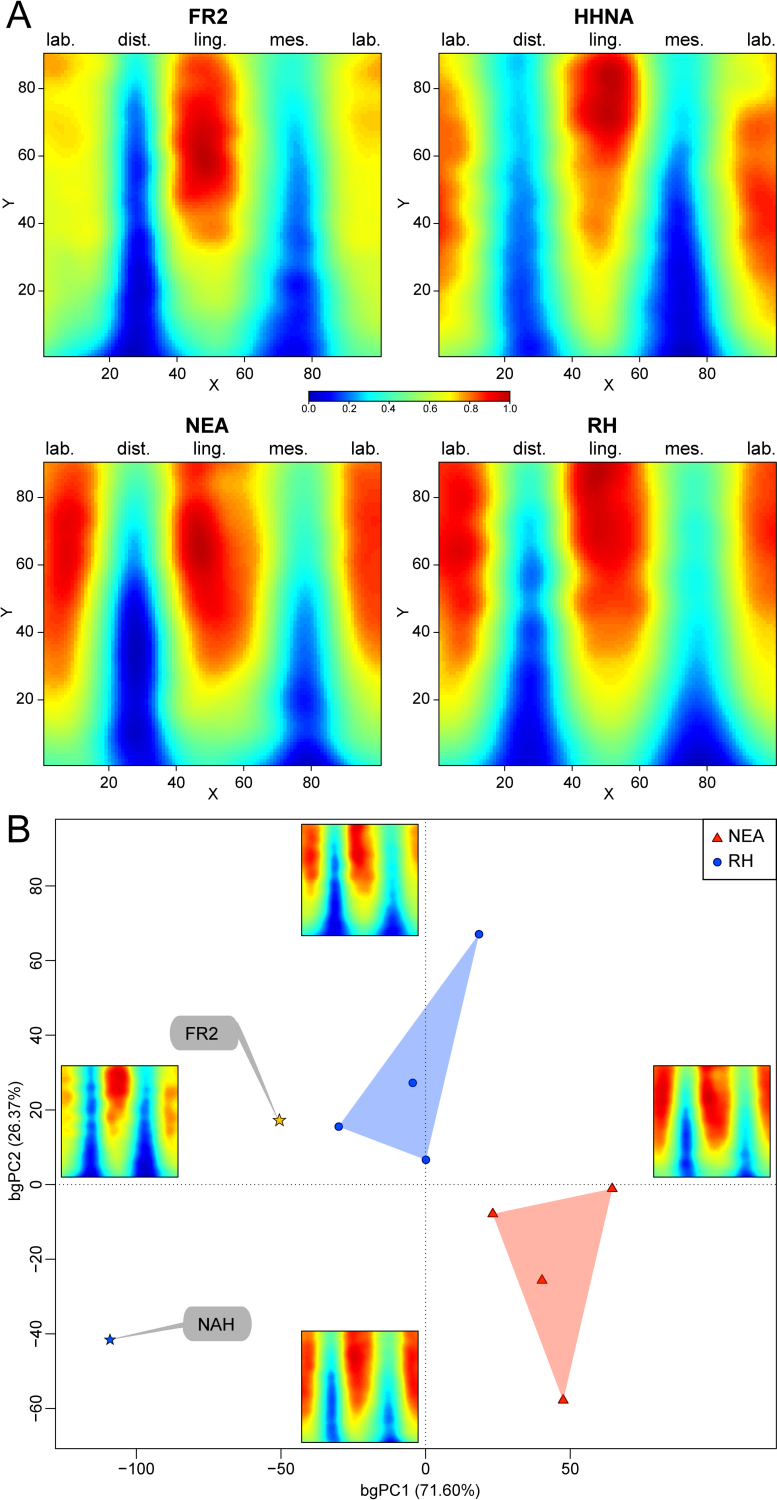
Standardized morphometric map of thickness variation for the root portion 15–85% of the LLI2 FR2 from Fontana Ranuccio (A) compared with that of the North African late Early-early Middle Pleistocene *Homo* (NAH) from Tighenif [59], as well as with the consensus maps representing four Neanderthal (NEA) specimens (KRD69, KRD71 from Krapina and the two LI2s from Regourdou) [60] and four extant humans (EH; original data). Each map is set within a grid made of 90 columns (X) running along the labial (lab.), distal (dist.), lingual (ling.) and mesial (mes.) aspects of the root and of 100 rows (Y). Relative thickness rendered by a chromatic scale increasing from dark blue (0) to red (1). The differences between FR2 and the comparative specimens/samples were assessed using between-group principal component analysis (B). Standardized morphometric maps representing the extreme conditions along bgPC1 and bgPC2 are illustrated at the end of the axes.

Together with the two consensus maps summarizing the information derived from four Neanderthal (from Krapina and Regourdou) and four extant human LI2s, respectively, and that of the fossil specimen from Tighenif, the standardized morphometric map of radicular dentine thickness topographic variation across the 15–85% root length portion of the FR2 incisor from Fontana Ranuccio is rendered in Fig 3A. In FR2, the virtually unzipped and unrolled chromatic-related root projection shows the thickest dentine in the 40–85% portion (proximal half) of the lingual aspect, while the thinnest tissue is found between approximately 15% and 80% of the distal and mesial aspects. A directly comparable distribution pattern is found in the specimen from Tighenif, but in this case labial dentine is much thicker. While a globally similar signature is also shared by the Neanderthal and extant human incisors examined in this study, compared to FR2 both consensus maps show wider thicker areas along the labial aspect, the thinnest dentine being confined within the c. 15–60% portion of the distal and mesial aspects. With this respect, the bgPCA analysis highlights the vertically extended areas of thinner dentine along the distal and mesial aspects shared by FR2 and Tighenif, while the Neanderthal pattern distinguishes from both Tighenif and the extant human pattern because of its globally thicker radicular dentine (Fig 3B).

### Premolar and molar lateral crown tissue proportions

**Fig 4:**
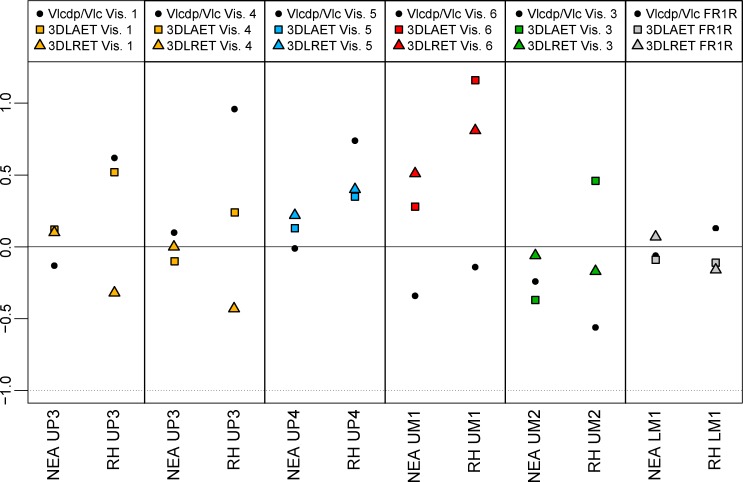
Adjusted Z-score graphs of the lateral crown tissue proportions variables (Vlcdp/Vlc, 3DLAET, 3DLRET) for the Vis. 1 and Vis. 4 UP3s, the Vis. 5 UP4, the Vis. 6 UM1, and the Vis. 3 UM2 from Visogliano, and for the FR1R LM1 from Fontana Ranuccio compared with similar evidence for Neanderthals (NEA) and extant humans (EH). The full line passing through the zero represents the average and the dotted lines correspond to the estimated 95% limit of variation expressed for each group (NEA and EH). See the Table 1 for details on the composition of the comparative samples.

Compared to the evidence from the assemblage of Tighenif, some late Middle-Late Pleistocene selected Neanderthal specimens, and a European extant human sample, the 3D lateral crown percent of lateral coronal volume that is dentine and pulp (Vlcdp/Vlc, in %) and the 3D lateral relative enamel thickness (3D LRET) of the premolar and molar crowns from Fontana Ranuccio (FR1R) and Visogliano (the upper premolars Vis. 1, Vis. 4 and Vis. 5 and the upper molars Vis. 6 and Vis. 3) are detailed in Table 2. The adjusted Z-score analysis (Fig 4) shows that the values of the three premolars from Visogliano more closely fit the Neanderthal than the extant human figures but, at least for the enamel thickness, fall within the variation range of the latter (Table 2). For the same variables, the upper molars Vis. 3 and Vis. 6 and the lower M1 from Fontana Ranuccio near the values found in the assemblage from Tighenif [59] but are still closer to the Neanderthal than to extant human estimates (Table 2, Fig 4). Notably for the 3D LRET, Vis. 6 is outside the range of variation of the comparative extant sample used in this study.

### Enamel-Dentine Junction (EDJ) morphology

**Fig 5:**
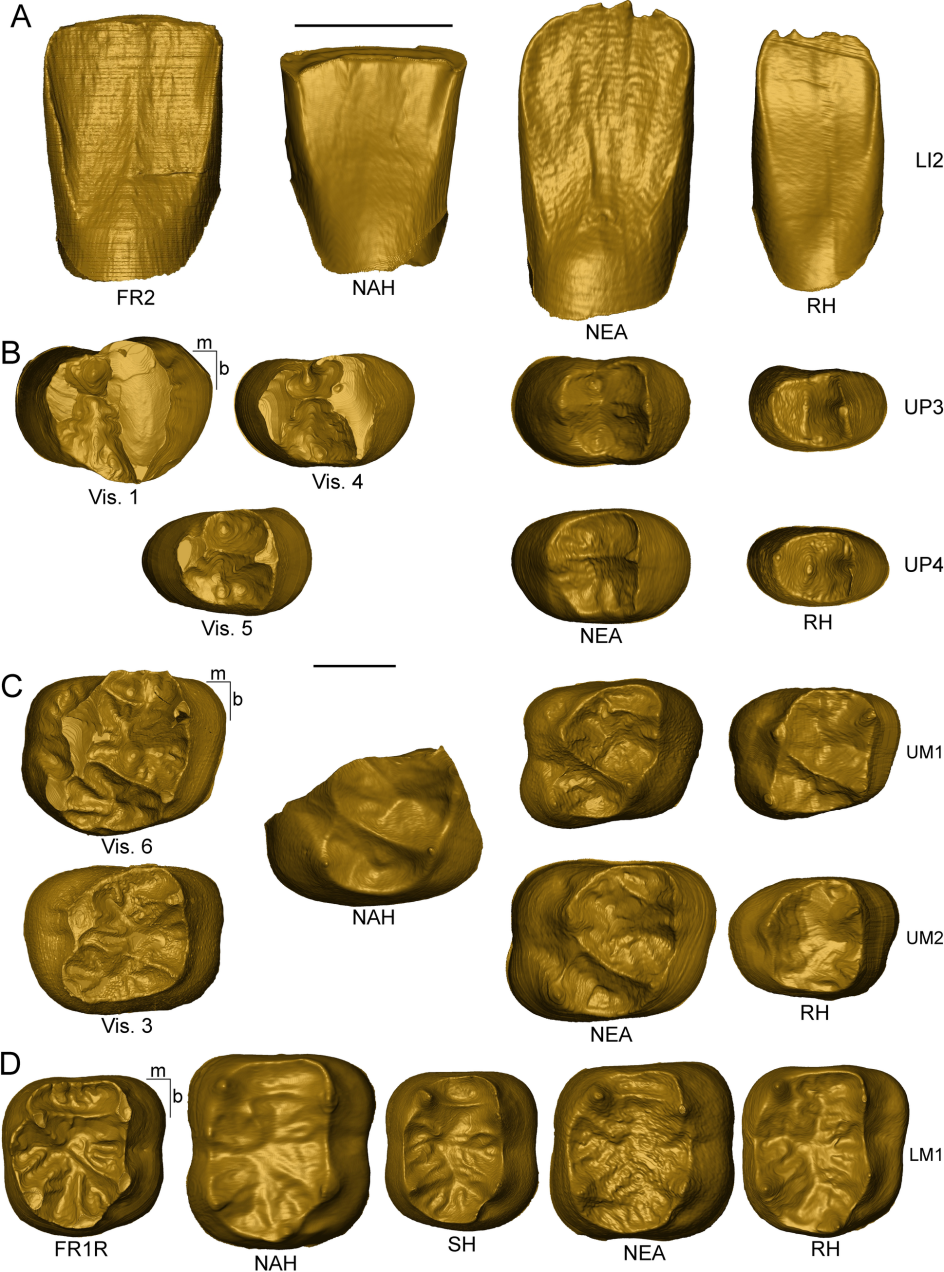
The enamel-dentine junction of the LLI2 FR2 from Fontana Ranuccio (A), of the upper premolars Vis. 1, Vis. 4, Vis. 5 (B) and upper molars Vis. 3, Vis. 6 (C) from Visogliano and of the lower molar FR1R (D) from Fontana Ranuccio compared with similar evidence for the North African late Early-early Middle Pleistocene *Homo* (NAH) from Tighenif (LRI2, URM1/2 and Tighenif 2 LM1) [59], a Sima de los Huesos human LM1 (AT-829) [41, 50], Neanderthals (NEA; KRD38, KRD41, KRD77, KRD90, KRD101, KRD165 from Krapina) [60] and extant humans (EH; original data). Independently from their original side, the LI2s (A), UP3s and UP4s (B), UM1s and UM2s (C) are shown as left crowns and the LM1s (D) are illustrated as right antimeres. Scale bars, 5 mm.

As illustrated in Fig 5, the virtual exploration of the EDJ allowed in most specimens from both sites the identification of a number of features partially masked at the outer enamel surface, or even completely erased by occlusal wear.

On its lingual aspect, the EDJ of the lower LI2 FR2 crown from Fontana Ranuccio exhibits a distinct tuberculum dentale (which is also visible at its outer surface; Fig 1), a feature also expressed in our Neanderthal sample [60], but not in the early Middle Pleistocene LRI2 from Tighenif [59], or in any extant human specimens available in our record (Fig 5A).

While incomplete because of extensive wear, the EDJ of the upper RP3 Vis. 1 reveals a large main buccal dentine horn (likely also including a smaller accessory distobuccal cusp, as suggested by the morphology of its pulp chamber; see below) and a smaller lingual horn (Fig 5B). The circular mesial fovea is enclosed by the complete mesial marginal and by transversal crests, while the larger distal fovea has a triangular shape. Even if its mesial marginal ridge is interrupted, this EDJ morphology recalls that of the smaller-sized ULP3 Vis. 4 specimen (Fig 5B).

The dentine morphology of the upper LP4 Vis. 5 is more symmetrical than seen in both UP3s from Visogliano, with a more centrally located transversal crest resulting in a well-developed mesial fovea, only slightly smaller than the distal one. These features are also seen in our Neanderthal upper premolar sample, while in extant humans the transversal crest is absent, leaving no distinction between the mesial and distal foveae, which typically form a unique occlusal basin (Fig 5B).

As also noted in some Neanderthal upper molars from Krapina (e.g., KRD101, KRD165; Fig 5C), the EDJs of Vis. 6 and Vis. 3 from Visogliano display: a weak expression of the Carabelli's trait, forming a slight shelf with some wrinkles running along the protocone (grades 4 and 3, respectively) [74]; an interrupted transverse ridge running mesially from the paracone to the mesial or central segment of the protocone; a complete oblique crest, even if slightly dipping in the middle in Vis. 3. With this respect, also the incomplete specimen from Tighenif preserves a well-developed, high oblique crest. The extant human UM1s and UM2s represented in our comparative sample show a simplified EDJ morphology with: low frequency of grade 4 and 3 Carabelli's trait (17.4% and 4.5%, respectively) [74]; no (or incomplete) transverse crest; low (and generally incomplete) oblique crest (Fig 5C).

The EDJ of the lower RM1 FR1R from Fontana Ranuccio shows a metaconulid-type tuberculum intermedium [75] and a high and continuous mid-trigonid crest with an interrupted distal trigonid crest (type 10) [41]. A mix of talonid crest patterns types 4 and 6 [50] is appreciable, with a small crest leaving the lingual segment of the distal trigonid area and reaching the lingual marginal crest slightly mesially from the tip of the entoconid (type 4). A discontinuous crest runs in distobuccal direction from the tip of the metaconid towards the apex of the hypoconulid (type 6) and there is also an accessory mesial entoconid ridge (Fig 5D). Compared to the morphology of the Italian specimen, the EDJ of the two LM1s of Tighenif 2 [50, 58] and the Neanderthal EDJs [32, 41, 76] also display a high and continuous mid-trigonid crest and sometimes either a type 4 or 6 talonid patterns, or a combination of both (for example, in the case of Tighenif 2). In the Atapuerca SH assemblage, the 14 specimens detailed so far reveal a continuous mid-trigonid crest (3/14 LM1s showing a type 10 pattern) [41], while no talonid crest pattern type 4 or 6 has been recorded in 22 lower M1s analysed for this feature [50]. Both uninterrupted mid-trigonid crest and complex type 4 or 6 talonid pattern are not frequent in extant humans [41, 50, 76].

It is noteworthy that, in addition to the morphological traits detailed above, all five isolated premolar and molar teeth from Visogliano and the lower first molar from Fontana Ranuccio show some accessory features expressed to an extent not noticed in the comparative materials used in this study. These include two- to three accessory cuspules lying on the mesial marginal ridge, as well as multiple accessory crests running from the respective marginal ridges towards the centre of the occlusal basin and vertical crests on the buccal or lingual aspect of the upper premolars and molars, respectively (Fig 5).

**Fig 6:**
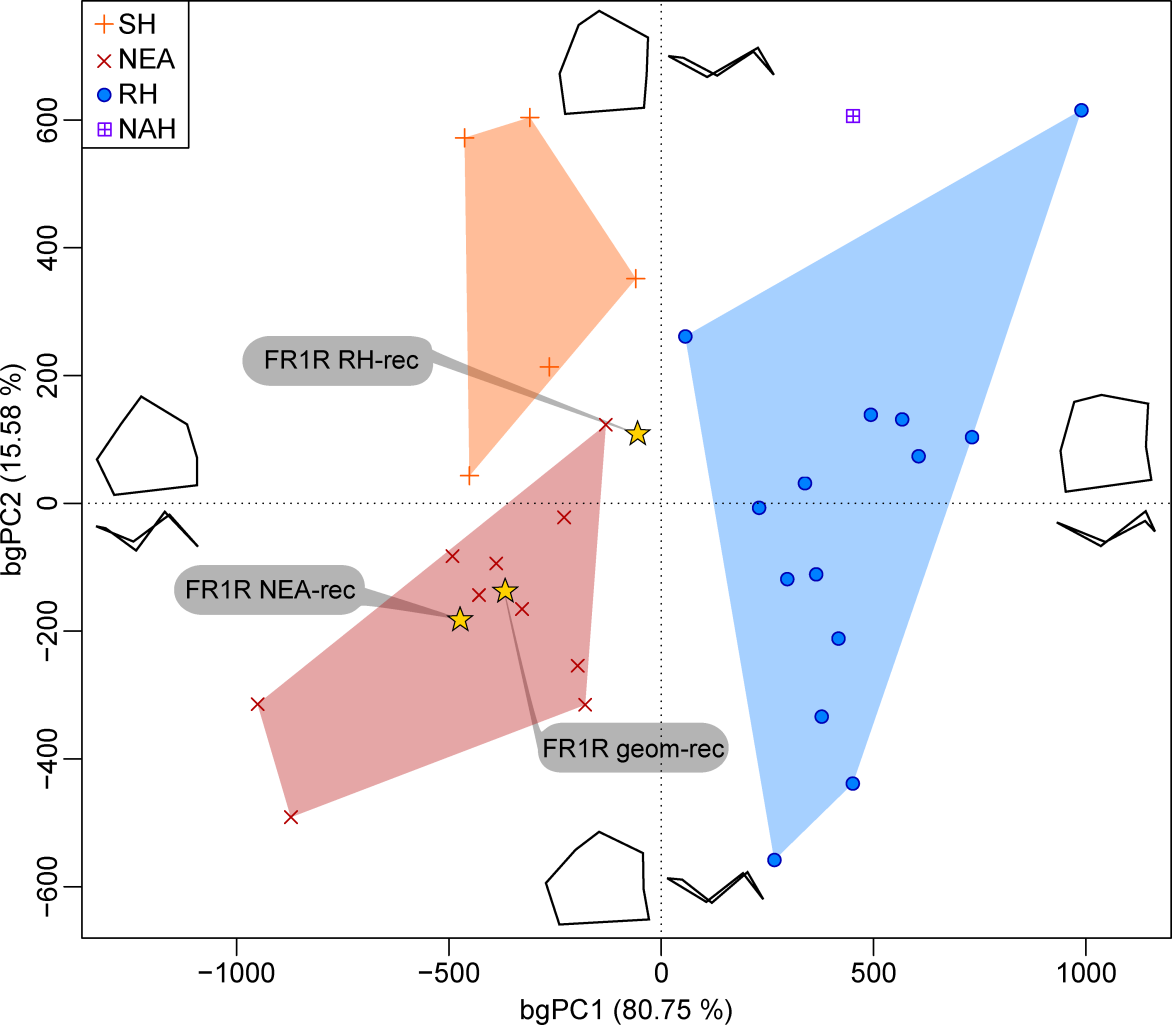
Between-group principal component analysis (bgPCA) of the Procrustes shape coordinates of the three reconstructions of FR1R LM1 EDJ and of the North African late Early-early Middle Pleistocene *Homo* (NAH) from Tighenif compared with the LM1s of Middle Pleistocene humans from Sima de los Huesos (SH), 10 Neanderthals (NEA) and 14 extant humans (EH). See the Table 3 for details on the composition of the comparative samples.

The bgPCA based on the EDJ Procrustes shape coordinates of the three reconstructions of the lower M1 from Fontana Ranuccio (FR1R geom-rec, FR1R NEA-rec and FR1R EH-rec; S1 Fig) are shown in Fig 6 together with the specimen from Tighenif 2, five LM1s from Sima de los Huesos, 10 Neanderthal and 14 extant human specimens (Table 3). The analysis of the between-group principal component scores in reference to the centroid size [77] indicates that there is no allometric signal in group separation along bgPC1 and bgPC2 (R^2^<0.05). In this comparative context, all three virtual reconstructions of the Visogliano's EDJ fall within (FR1R geom-rec and FR1R NEA-rec) or immediately close to (FR1R EH-rec) the Neanderthal range (characterized by relatively high dentine horns and a mesiodistally elongated trigonid), and set apart from the variation shown by Sima de los Huesos (displaying an overall pattern similar to that expressed by the Neanderthal sample used here, but with a more mesially placed metaconid), as well as from the extant human condition (associated to a lower EDJ topography and to a more squared trigonid). There is also a chronospatial heterogeneity of the Neanderthal signal, the earliest specimen from Ehringsdorf being the closest to the Sima de los Huesos remains along bgPC2, whereas the assemblage from La Chaise-de-Vouthon falls in a more negative part of the axis. The LM1 of the late Early-early Middle Pleistocene Tighenif 2 lies on the positive space of bgPC1, close to the extant human condition and distinct from the Neanderthal-like pattern.

### Pulp cavity morphology

**Fig 7:**
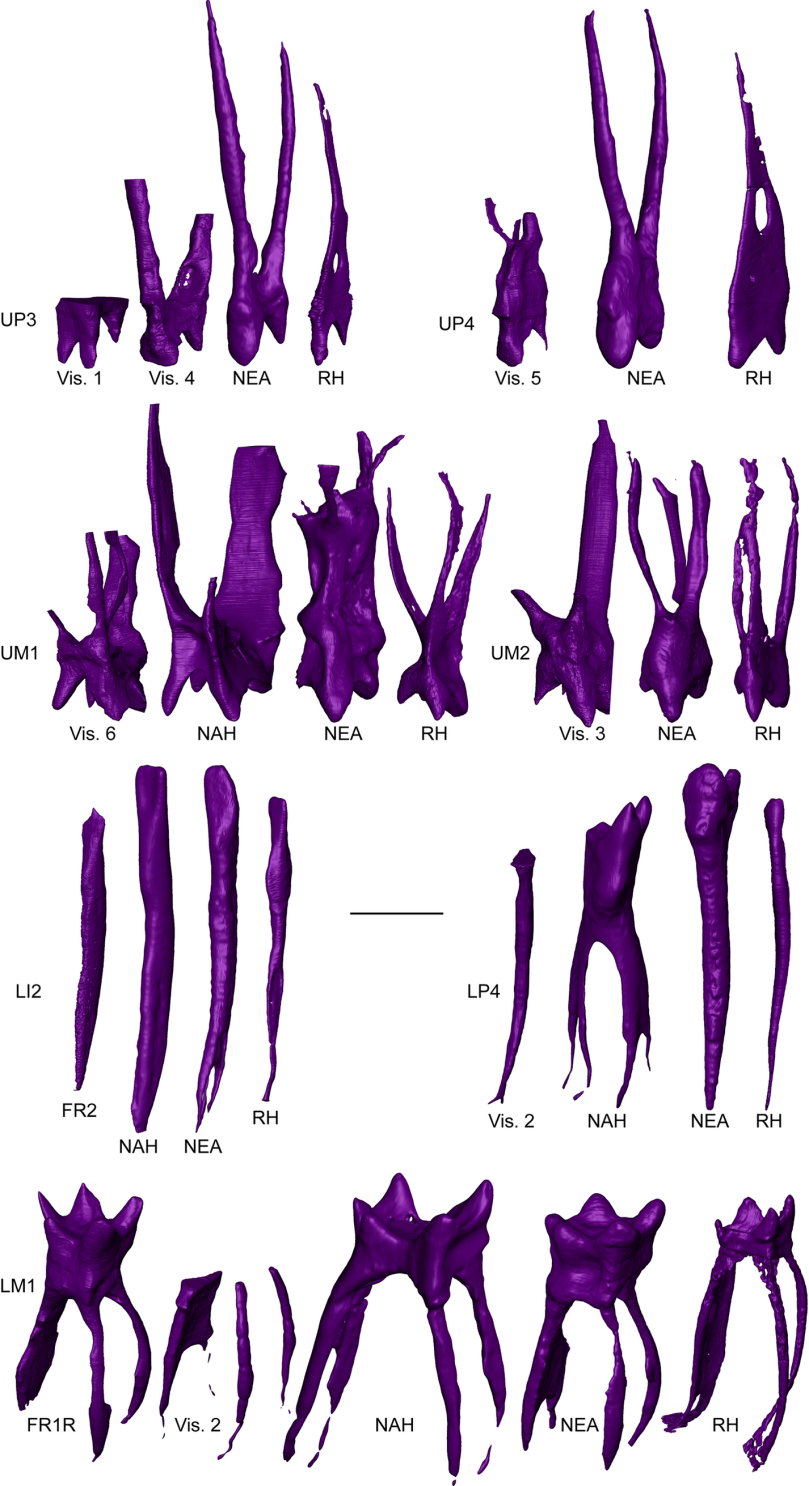
Virtual rendering of the pulp cavity of the UP3 Vis. 1, the UP4s Vis. 4 and Vis. 5, the UM1 Vis. 6, the UM2 Vis. 3, the LI2 FR2, the LP4 Vis. 2 and the LM1s FR1R and Vis. 2 compared with similar evidence for the North African late Early-early Middle Pleistocene *Homo* (NAH) from Tighenif (isolated URM1/2, isolated LRI2 and Tighenif 2 LP4 and LM1) [59], Neanderthals (NEA; KRD30, KRD41, KRD45, KRD71, KRD 79, KRD136, KRD165 from Krapina) [60] and extant humans (EH; original data). All specimens are shown as right antimeres. The LI2s are displayed in mesio-lingual view, whereas the post-canine teeth are illustrated in mesio-buccal view. Scale bar, 5 mm.

The pulp cavity of the FR2 lower lateral incisor from Fontana Ranuccio is labiolingually compressed in the crown portion and mesiodistally flattened along the root. A similar morphology is seen in the larger LRI2 from Tighenif [59], while the Neanderthal LI2s examined here have a more cylindrical root canal. With this respect, the cavity root portion is commonly less flattened in extant human lower incisors (Fig 7).

The URP3 Vis.1 from Visogliano presents a peculiar chamber morphology, with two buccolingually flattened buccal horns projecting in occlusal direction and a smaller, parallel, conical lingual horn. In the upper premolars Vis. 4 (P3) and Vis. 5 (P4) there are only two pulp horns, a buccal and a lingual, also buccolingually flattened and conic-shaped, as well as two main root canals (even if the buccal canal in Vis. 5 tends to diverge apically). The Neanderthal upper premolar cavities examined in this study are similar to the morphology seen in Vis. 4 and Vis. 5, even if the buccal horn is usually less compressed. Conversely, extant humans typically display two conical buccal and lingual horns, a more mesiodistally compressed chamber and fused root canals (Fig 7).

The pulp chamber of the URM1 Vis. 6 and of the URM2 Vis. 3 show four large conical horns, all diverging and projecting externally. Both teeth display a mesiobuccal, a distobuccal and a lingual main root canal (even if the mesiobuccal canal of Vis. 6 is bifurcated). A similar pulp morphology is found in the URM1/2 from Tighenif [59], but such pattern can also be observed in Neanderthal UM1s and UM2s, even if in this case the pulp horns are more bulky and there is a relative reduction of the hypocone horn. Extant humans are set apart, having more occlusally oriented pulp horns and generally expressing no hypocone horn (or a very small one) and closely set root canals (Fig 7).

The LRP4 root fragment included in the partial mandible Vis. 2 from Visogliano exhibits a slightly mesiodistally compressed single pulp canal with small apical canaliculi (Fig 7). This differs from the more primitive molarized morphology in the Tighenif 2 specimen [59], but more closely resembles the Neanderthal and extant human conditions.

Finally, the pulp cavity of the LRM1 FR1R from Fontana Ranuccio displays five sharp horns of comparable size, surmounting a large, high chamber with three main root canals (a mesiobuccal, a mesiolingual and a larger, mesiodistally flattened distal one) as seen in Vis. 2 (Fig 7). While in this case the Tighenif 2 and the extant human lower first molars show proportionally smaller and more constricted pulp chambers, Neanderthal LM1s, which often express a variable degree of taurodontism [32, 35], more closely resemble the proportions displayed by FR1R (Fig 7).

## Discussion

The European Middle Pleistocene human record is, to the exception of some exceptional sites like Atapuerca Sima de los Huesos, in Spain [26] and Caune de l’Arago at Tautavel, in France [78], still limited to a few chronospatially scattered localities [25, 79]. Besides random factors due to fossilization and discoveries of fossil sites, this discontinuous distribution could also reflect population dynamics in relation to climatic fluctuations determining phases of repeated colonization during the interstadial and interglacial periods followed by local extinctions/retractions during the harsher glacial periods [80]. In particular, the MIS 12, when the human groups from Visogliano and Fontana Ranuccio lived, was among the coldest of the Pleistocene epoch [81, 82] and the related paleoecological conditions have likely impacted human settlements dynamics. Recent morphological and paleogenetical studies suggest higher rates of population size fragmentation and bottlenecks among Eurasian groups during the late Early and early Middle Pleistocene [19, 22, 83]. In this context, an “ebb and flow” demographic model, with a central area of dispersals located somewhere between Eastern Europe and Asia continuously inhabited by a “source population”, could parsimoniously explain the evolution of European Middle Pleistocene hominins and the apparent non- linear trajectory towards Neanderthals [84–86]. Indeed, one of the most disputed questions concerns the taxonomic identity of the humans groups that were in Eurasia during this period. Until recently, most of the Middle Pleistocene human fossils found in Europe (and even sometimes in Africa and Asia) were gathered together into groups referred as “archaic modern humans” or “*Homo heidelbergensis* s.l.” [87, 88]. But their relationships with earlier groups attributed to *H*. *antecessor* and *H*. *erectus/ergaster* and with the successive Neanderthals and modern humans, as well as the taxonomic unity vs. diversity of these European Middle Pleistocene populations are increasingly debated [25, 79, 89–91]. A recent comparison between the Atapuerca SH and Arago dental remains showed that while the teeth of the former assemblage are practically indistinguishable from European Neanderthal teeth, those of the latter exhibit a suite of plesiomorphic features alongside some characteristic Neanderthal traits, suggesting the existence of more than one hominin lineage in these two geographically and chronologically close sites [92].

Our endostructural analyses of the dental remains from the Italian sites of Fontana Ranuccio and Visogliano brings new evidence to characterize the extent of the morphological variability of the Southern European Middle Pleistocene human record [22].

While the specimens from Fontana Ranuccio are relatively well preserved and more easily recognized [10], the identification of some of the more fragmentary elements from Visogliano remains contentious, such as the tooth fragments Vis. 7, Vis. 8 and Vis. 9 (not analyzed here, see [17]) and the maxillary crown Vis. 1. This latter was previously tentatively recognized as a left third premolar [93] or as a right third molar [17]. More recently, it was proposed that this tooth could represent an upper right third premolar [11, 18]. There is now more evidence to support this latter hypothesis. The crown shape of Vis. 1 is close to the UP3 variation seen in the Middle Pleistocene assemblage from Atapuerca SH [94], and is compatible with the variability encountered by our recent human sample. In addition, the occlusal wear pattern of Vis. 1 (with a large patch of dentine on the largest cusp, that we identified as the buccal cusp, and only a small island on the other one, the lingual cusp) is similar to mirrored image of the ULP3 Vis. 4. This suggests that Vis 1 represents an URP3. Vis. 1 EDJ also shows two foveae (anterior and posterior) separated by a transverse crest as seen in the two other premolars from the same site, Vis. 4 and Vis. 5, but not the many occlusal crests and ridges visible in the URM1 Vis. 6 and in the ULM2 Vis. 3 (Fig 5B). The three-horned pulp chamber of Vis. 1, even if possibly suggesting the presence of three cusps, is more similar in shape to those of Vis. 4 and Vis. 5 (with the buccal horns having a buccolingually compressed, spatulate-like shape and a parallel lingual one which is conical) rather than to those of Vis. 3 and Vis. 6 (that display conical buccal and lingual pulp horns that diverge from each other). Based on this new record, we reassess the serial position of Vis. 1 as more likely representing an URP3.

The internal tooth structural organization of the Visogliano and Fontana Ranuccio assemblage reveals a Neanderthal-like signal, also resembling some aspects seen in the assemblage from Atapuerca SH (notably, for the non-metric features of the LM1) and differing from the recent human condition. More precisely, the LLI2 FR2 exhibits thin labial enamel, as typically found in the Neanderthal lineage [32, 33, 49, 95]. However, the peculiar thin dentine root of FR2, even proportionally thinner than measured in our comparative modern sample and approaching in some respects the pattern seen in the isolated LRI2 from Tighenif, differs from the typical Neanderthal condition. This result confirms the interest of extracting subtle structural variation in root architecture in extant and fossil human teeth, which has the potential of revealing functionally/biologically-related differences in the local distribution of dentine thickness [61]. Compared to the extent human condition, the thicker labial and lingual root aspects of the Neanderthal LI2s (Fig 3) could be related to allometry (Neanderthal incisors tend to have larger roots with a more developed pulp cavity) [96, 97], to different secretory patterns of secondary dentine (Neanderthals and modern humans apparently deposit secondary dentine in different locations of the pulp cavity) [35] and/or to function (as Neanderthals, including the specimen Regourdou 1 considered in this study, commonly used their anterior teeth as a ‘third hand’) [98, 99].

Regarding the post-canine dentition of the Italian Middle Pleistocene remains considered in this study, it is characterized by thin lateral enamel, close to the late Early-early Middle Pleistocene specimens from Tighenif and similar to that of Neanderthals, but again different from the thicker modern human condition. It is noteworthy that, like the LM1 FR1R from Fontana Ranuccio, the penecontemporaneous LM3 Arago 106 from Tautavel exhibits a lateral enamel thickness index (3DLRET: 9.40) that more closely approximates the Neanderthal average (3DLRET: 9.59) than the recent human condition (3DLRET: 8.82) [49].

In the LLI2 FR2, a well-developed tuberculum dentale is well-expressed at both outer enamel and EDJ levels, as frequently found in the SH assemblage (79.0%) and in Neanderthals (71.4%), but not in recent humans (19.9%) [45, 100]. The EDJ of the Visogliano and Fontana Ranuccio teeth shows an overexpression of the main and accessory features. In Vis. 6 and Vis. 3, it cumulates a combination of traits recently identified by Martin and collaborators [42] as more developed in Neanderthals, like the intermediate and minor post-paracone tubercle in the UM1 and UM2 respectively, a type I crista obliqua in both upper molars from Visogliano (even if type II is more frequent in early Neanderthal UM2s), and the lingually-set hypocone. The same is true in the LM1 FR1R, with the centrally-placed metaconid, a continuous mid-trigonid crest with an incomplete type 10 distal trigonid crest pattern [41, 101]), and an intermediate type 4–6 talonid crest pattern [50]. Conversely, the molars of the penecontemporaneous Balanica mandible BH-1 [47], from Serbia, express no clear Neanderthal traits, with a low and discontinuous mid-trigonid crest corresponding to type 4 never recorded in the Atapuerca SH or Neanderthal LM1s, but frequent in modern humans [41], and no obvious talonid crest pattern [47]. This condition is similar to that reported for the Mauer lower molars [76, 102]. Interestingly, the 300–400 ka deciduous and permanent dental sample from Qesem Cave [102, 103], in Israel, also displays a combination of Neanderthal structural features, including the crown and cervical outline shape, the position of the cusps (notably the more inward placement of the lingual dentine horns apex), the high topography of the EDJ, a continuous and high mid-trigonid crest type 10 (not type 12; contra [102]), and a type 6 talonid crest patterns in M_2_-QC12 [102]. Noteworthy, the latter specimen lacks the hypoconulid, which is unusual in the early to mid-Middle Pleistocene record, except in a few cases from the Sangiran Dome, in Java [104], and in the Atapuerca SH assemblage [45].

The Chinese teeth from Longtan Cave estimated to 412±25 ka [105], also share a number of characteristics with the Visogliano and Fontana Ranuccio specimens: large external crown dimensions and robust roots (with three-rooted upper premolars and low-bifurcated lower molar roots), as well as a complex internal structural morphology. This includes the presence of vertical wrinkling on the buccal aspect of the upper premolars and on the mesiolingual corner of the upper molars EDJ (forming a particular expression of the Carabelli’s trait [74]) and numerous accessory ridges in the occlusal basin of the EDJ of premolars and molars (even if the wrinkling pattern is more developed in the Asian specimens from Hexian and Zhoukoudian [105]). But the Italian Middle Pleistocene specimens also show proportionally higher EDJ topography (with a deeper occlusal basin and more prominent cusp dentine horns) and more developed pulp cavities (with taurodontic-like pulp chambers resembling those of Neanderthals). However, the nature of these similarities (e.g., homologies or appearance of similar autapomorphies in both groups) and differences (autapomorphies vs. synapomorphies), and thus of the longitudinal evolutionary dynamics across Eurasia, remain to be clarified [85, 86].

## Concluding remarks

Recent development of sequencing techniques allowed recovering mitochondrial DNA and significant portions of the nuclear genome of Middle Pleistocene human remains from Atapuerca Sima de los Huesos [20, 21, 106]. Results revealed more complex population interactions through time and space than previously established. In particular, nuclear genomic data indicate closer relationships between the SH hominins and European Neanderthals than with the Siberian Denisovans [21]. In light of these advances, it appears that paleogenetic research has the potential to shed new light on the paleoanthropological conundrum of the *H*. *heidelbergensis* status and to make clearer the complex phylogenetic relationships of the late Early to Middle Pleistocene Eurasian populations [25, 79, 85]. Both genetic [83, 107] and morphological evidence [108] now suggest an early divergence between Neanderthals and modern humans, likely around the Early-Middle Pleistocene transition.

Even if limited to two geographically scattered and small samples, our comparative study of the inner structural organization of the tooth remains from Fontana Ranuccio and Visogliano provides additional evidence supporting such scenario. Our results show that the overall Neanderthal morphological dental template was preconfigured at least 430 to 450 ka ago, which is in accordance with some cranial studies [19, 22, 24, 25]. However, the existence of early to mid-Middle Pleistocene dentognathic specimens like Mauer 1 and Balanica BH-1, showing none or few Neanderthal-derived traits in their inner tooth structure [47, 76, 102], also indicates that other human groups, not necessarily related to the Neanderthal lineage, may have existed in Eurasia around the same period.

## Supporting information

S1 Fig Methods for reconstructing the worn dentine horn apices of the LRM1 FR1R specimen from Fontana Ranuccio. The first method followed a 2-3D geometric approach (FR1R geom-rec) based on the sections intersecting the centre of the dentine horns followed by interpolation (A). The 3D rendering of the reconstructed EDJ surface of FR1R geom-rec is compared with two independent reconstructions (second method) respectively based on the superimposition of a Neanderthal (Fossellone 3) [1,2] (FR1R NEA-rec) and of an extant human LM1 crown (FR1R EH-rec) used as templates (B). Scale bar, 5 mm.
(DOCX)

S2 Fig Position of the seven landmarks placed on the EDJ of an extant human LM1 showed in occluso-buccal view. b, buccal; d, distal; l, lingual; m, mesial. Scale bar, 5 mm.
(TIF)
