# Supplementary material for: The Middle Pleistocene (MIS 12) human dental remains from Fontana Ranuccio (Latium) and Visogliano (Friuli-Venezia Giulia), Italy. A comparative high resolution endostructural assessment
Source: PLoS One. 2018 Oct 3;13(10):e0189773. doi: 10.1371/journal.pone.0189773 (PMC6169847; doi:10.1371/journal.pone.0189773)
Supplement: S1 Fig — The first method followed a 2-3D geometric approach (FR1R geom-rec) based on the sections intersecting the centre of the dentine horns followed by interpolation (A). The 3D rendering of the reconstructed EDJ surface of FR1R geom-rec is compared with two independent reconstructions (second method) respectively based on the superimposition of a Neanderthal (Fossellone 3) [1,2] (FR1R NEA-rec) and of an extant human LM1 crown (FR1R EH-rec) used as templates (B). Scale bar, 5 mm. (DOCX) [file pone.0189773.s001.docx]

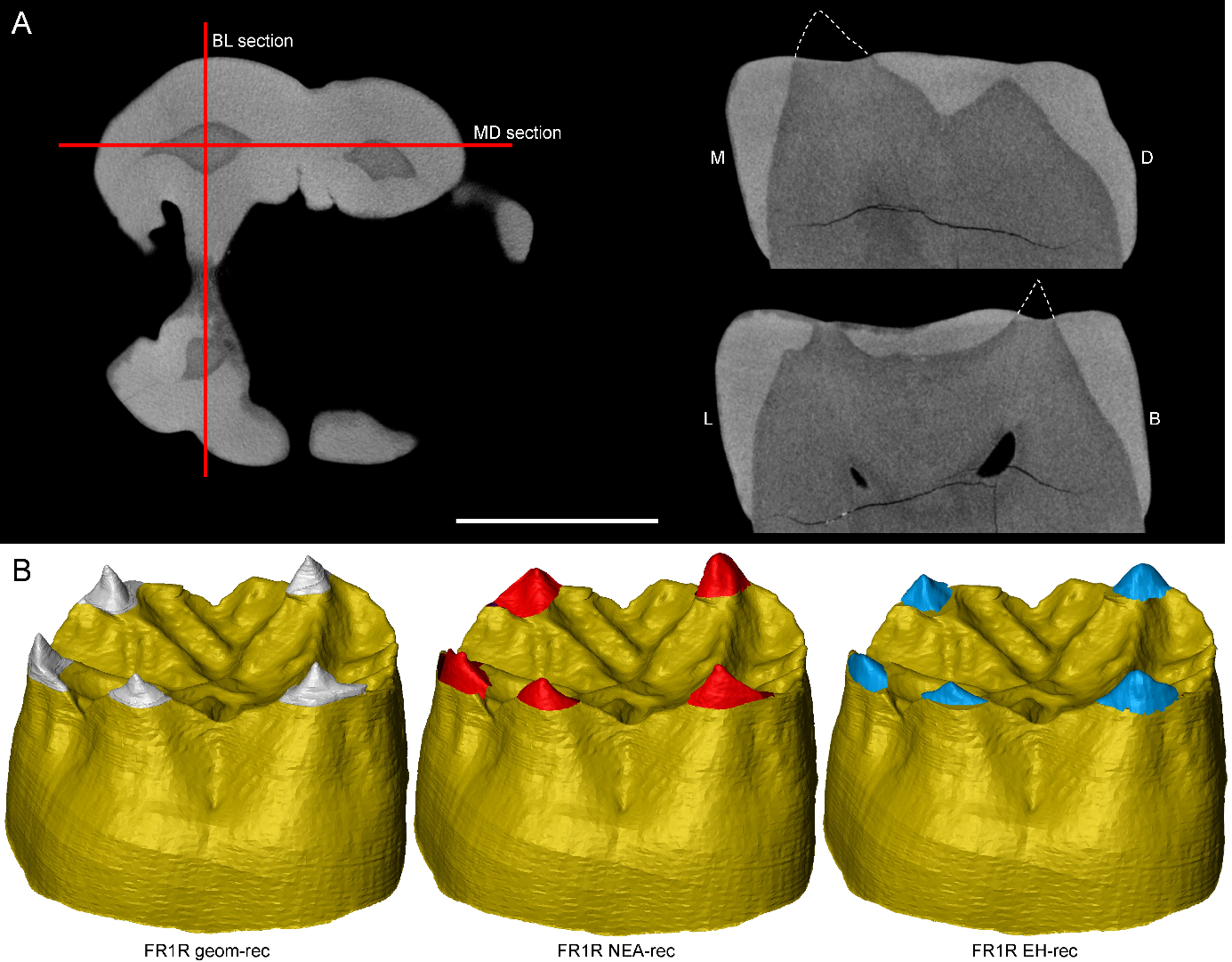


**S1 Fig.** **Methods for reconstructing the worn dentine horn apices of the LRM1 FR1R specimen from Fontana Ranuccio**. The first method followed a 2-3D geometric approach (FR1R geom-rec) based on the sections intersecting the centre of the dentine horns followed by interpolation (A). The 3D rendering of the reconstructed EDJ surface of FR1R geom-rec is compared with two independent reconstructions (second method) respectively based on the superimposition of a Neanderthal (Fossellone 3) [1,2] (FR1R NEA-rec) and of an extant human LM1 crown (FR1R EH-rec) used as templates (B). Scale bar, 5 mm.

**References**

**1.** Mallegni F. Human remains (Fossellone 3; ex Circeo 4) referable to Homo s. neanderthalensis from Fossellone (Monte Circeo, Latium, Italy). Riv Anthropol. 1992; 70: 217-227.

**2.** Tuniz C, Bernardini F, Bondioli L, Coppa A, Dreossi D, Macchiarelli R, et al. X-ray microtomographic-based structural analysis of the dental remains from the Mousterian and Aurignacian levels of the Fossellone cave, Latium, Italy. Proc. ESHE. 2014; 3: 163 (abstract).
